# Isolation and genetic characterization of a relapsing fever spirochete isolated from *Ornithodoros puertoricensis* collected in central Panama

**DOI:** 10.1371/journal.pntd.0009642

**Published:** 2021-08-16

**Authors:** Sergio E. Bermúdez, Brittany A. Armstrong, Lillian Domínguez, Aparna Krishnavajhala, Alexander R. Kneubehl, Sarah M. Gunter, Adam Replogle, Jeannine M. Petersen, Job E. Lopez

**Affiliations:** 1 Departamento de Investigación en Entomología Médica, Instituto Commemorativo Gorgas de Estudios de la Salud, Ciudad de Panama Panama; 2 Department of Pediatrics, The National School of Tropical Medicine, Baylor College of Medicine, Houston, Texas, United States of America; 3 Department of Molecular Virology and Microbiology, Baylor College of Medicine, Houston, Texas, United States of America; 4 Centers for Disease Control and Prevention, Division of Vector-Borne Diseases, Bacterial Diseases Branch, Fort Collins, Colorado, United States of America; Rocky Mountain Laboratories, NIAID, NIH, UNITED STATES

## Abstract

Tick-borne relapsing fever (TBRF) spirochetes are likely an overlooked cause of disease in Latin America. In Panama, the pathogens were first reported to cause human disease in the early 1900s. Recent collections of *Ornithodoros puertoricensis* from human dwellings in Panama prompted our interest to determine whether spirochetes still circulate in the country. *Ornithodoros puertoricensis* ticks were collected at field sites around the City of Panama. In the laboratory, the ticks were determined to be infected with TBRF spirochetes by transmission to mice, and we report the laboratory isolation and genetic characterization of a species of TBRF spirochete from Panama. Since this was the first isolation of a species of TBRF spirochete from Central America, we propose to designate the bacteria as *Borrelia puertoricensis* sp. nov. This is consistent with TBRF spirochete species nomenclature from North America that are designated after their tick vector. These findings warrant further investigations to assess the threat *B*. *puertoricensis* sp. nov. may impose on human health.

## Author summary

Tick-borne relapsing fever (TBRF) is an often misdiagnosed neglected tropical disease primarily impacting those in resource limited settings. Most species are transmitted by argasid ticks, but ixodid ticks and the human body louse are also competent vectors. Infection of TBRF spirochetes is challenging to diagnose because argasid ticks are rapid feeders and rarely spotted on the patient. Moreover, given the nonspecific manifestation of disease and poor clinical awareness, TBRF is overlooked. In Central America, TBRF was studied in the Canal Zone of Panama until the 1930s. Over the last 10 years argasid ticks have been reported to colonize human dwellings in the country but it has remained vague if the disease persists. In this study, we demonstrate the transmission of TBRF spirochetes from field collected ticks and the laboratory isolation and genetic characterization of the species. These findings suggest that TBRF spirochetes continue to circulate in Panama and should prompt health care providers to consider this disease when patients present with a fever of unknown origin. Furthermore, with the laboratory isolation of this bacteria, molecular diagnostic tools can be developed to improve epidemiological studies of TBRF spirochetes in Central America.

## Introduction

In the Americas, tick-borne relapsing fever (TBRF) was first reported to cause human disease in Panama in 1909 when spirochetes were detected in the blood of Canal Zone workers [1]. Blood samples from infected workers were used to inoculate monkeys, rats, and mice, which caused a progression and relapses mimicking that observed in humans. These findings suggested that the spirochetes were pathogenic. In 1921, human susceptibility was validated through the infection and monitoring of disease progression of three soldiers [2]. One was infected by needle inoculation with spirochete infected rat blood, the other by inoculation of triturated field collected ticks, and the third by tick bite. Outside of these early studies, very little has been done to investigate TBRF in Central America.

Throughout Central and South America there has been confusion regarding the distribution and speciation of argasid ticks that transmit TBRF spirochetes. Early investigations designated *Ornithodoros talaje* as the vector of TBRF spirochetes throughout the continent. *Ornithodoros talaje* was originally described in 1849 from Guatemala, and in 1921 Bates, Dunn, and St. John reported that the species was the causative vector of TBRF in Panama [2]. However, as additional work was performed by Dunn he renamed *O*. *talaje* and designated the ticks as *Ornithodoros venezuelensis*, which is also synonymous to *Ornithodoros rudis* [3]. Further complicating the matter is the distribution of *Ornithodoros puertoricensis* in Panama because this species is indistinguishable from *O*. *talaje* at the nymphal and adult stage [4]. Consequently, Hoogstraal and others have questioned the validity of most records reporting the distribution of *O*. *talaje* prior to 1950 because the morphological studies were based on adult ticks [5]. Therefore, it is not entirely clear as to what *Ornithodoros* species are vectors of TBRF spirochetes in Central and South America.

*Ornithodoros rudis*, *O*. *talaje*, and *O*. *puertoricensis* are known to parasitize humans and while most work has focused on the former two *Ornithodoros* species, recent studies demonstrated the continued circulation of *O*. *puertoricensis* in Panama [6]. In 2015, argasid ticks were collected in human dwellings in Ancón and the province of Colón where homeowners reported “insect bites.” Morphological characterization indicated the collection of *O*. *puertoricensis* and sequencing the 16s ribosomal RNA (*rrs*) gene further supported these observations. While *Borrelia* DNA was not detected in these specimens, the identification of *O*. *puertoricensis* in human dwellings indicated a potential public health threat and warranted further investigation.

In this current study we expanded field collection efforts to determine whether TBRF spirochetes circulate in central Panama, formerly known as the Canal Zone. Field studies to collect *O*. *puertoricensis* ticks were initiated at sites around Gamboa and the Ancón community of the City of Panama. In the laboratory, field collected ticks were fed on mice and we successfully isolated spirochetes from murine blood in culture medium. Genetic typing was performed to determine the relatedness of these spirochetes to known Western Hemisphere TBRF *Borrelia*. Our findings indicate that TBRF spirochetes continue to circulate in Panama and this work will guide future efforts to determine the public health relevance.

## Methods

### Ethics statement

Animal studies were approved by the Institutional Animal Care and Use Committee at Baylor College of Medicine. The laboratory animal program adheres to guidelines established by the Association for Assessment and Accreditation of Laboratory Animal Care and the National Institutes of Health Office of Laboratory Animal Welfare. Animal husbandry was provided by trained animal care technicians and veterinary staff.

### Tick collections

From 2016–2018, we conducted field studies centered on collecting ticks in central Panama. One field site was in Gamboa where collection efforts were focused on dens in a residential area. In the Ancón community of the City of Panama efforts were centered at four sites in Summit Botanical Gardens and Zoo. Two collection methods were implemented to capture argasid ticks. In the first approach, dry ice was used as a source of carbon dioxide to lure ticks from dens. The second approach utilized an aspirator to remove debris from dens through suction, and we manually sifted through the material using a series of #10 - #40 mesh strainers. Specimens collected from a given den were placed in 15 ml ventilated centrifuge tubes and each population was kept separate.

### Animal studies and serology

Cohorts of five to 10 field collected ticks were first fed on Institute of Cancer Research (ICR) mice (an outbred colony of Swiss origin maintained at Baylor College of Medicine). To determine if ICR mice were infected with spirochetes, ~2.5 μl of blood was collected for 10 consecutive days and analyzed by dark field microscopy. After 30 days the animals were exsanguinated and antibody responses were evaluated by immunoblotting, as described below. Remaining live ticks from one location were subsequently allowed to feed on a MyD88^-/-^ mouse. When spirochetes were visualized in the blood, the animal was exsanguinated and serum was used to inoculate modified Barbour-Stoenner-Kelly (mBSK-R) medium [7]. Spirochetes were passaged into 50 ml culture tubes for DNA isolation.

Immunoblotting was performed to assess serological responses of ICR mice that were fed upon by *O*. *puertoricensis*. Protein lysates from 1 x 10^7^ *Borrelia turicatae* 91E135 were used for SDS-PAGE and proteins were transferred to polyvinylidene fluoride (PVDF) membranes, as previously described [8]. Serum samples were diluted 1:200 and used to probe PVDF membranes. The secondary molecule used was Protein G-HRP (Life Technologies, Carlsbad, CA, USA) diluted 1:4,000. Antibody responses were detected with the ECL Western Blotting Detection Reagent (GE Healthcare, Buckinghamshire, UK) and immunoblots were imaged with a ChemiDoc MP (Bio-Rad, Hercules, California, USA).

### Molecular analysis of field collected ticks

In the laboratory, tick identity was confirmed by molecular methods. We extracted DNA from at least five specimens from each den using the DNeasy Blood & Tissue kit (Qiagen, Hilden, Germany). PCR was performed on the *rrs* locus as previously described with the following primer pairs 16s + 1: CTGCTCAATGATTTTTTAAATTGC and 16s-1: CCGGTCTGAACTCAGATCATGTA [6,9]. Amplicons were Sanger sequenced by GeneWiz (South Plainfield, New Jersey, USA) and the data were assembled and trimmed using Vector NTI software (Life Technologies, Carlsbad, CA, USA).

### Genetic analysis of the *Borrelia* isolated from *O*. *puertoricensis*

Pulsed-field electrophoresis and a phylogenetic analysis were performed to characterize the novel *Borrelia*. Spirochetes passaged no more than five times in medium were grown in 50 ml culture tubes and genomic DNA was isolated by phenol-chloroform extraction, as previously described [10]. To evaluate plasmid content, pulsed-field electrophoresis was performed using a PPI-200 Programmable Power Inverter (MJ Research, Watertown, MA), as previously reported with minor modifications [11]. Genomic DNA (1 μg) from *B*. *turicatae* 91E135, *Borrelia parkeri* SLO, *Borrelia hermsii* DAH, *Borrelia anserina* BA2, and the novel *Borrelia* were electrophoresed in 1% agarose gels with 0.5x TBE buffer (45 mM Tris, 45 mM boric acid, and 1.8 mM EDTA) for 15 min at 100 V and run on Program 3 for 40 hr at 90 V.

We also amplified *Borrelia* DNA gyrase subunit B gene (*gyrB*), flagellin gene (*flaB*), *rrs*, and the intragenic spacer (IGS) locus by polymerase chain reaction (PCR). Table 1 lists the primer sequences used, and PCR was performed as previously described [11]. PCR amplicons were purified using QIAquick PCR Purification Kit (Qiagen, Hilden, Germany), and were Sanger sequenced by GeneWiz (South Plainfield, New Jersey, USA). Chromatograms were visualized and the ends trimmed using Vector NTI software (Life Technologies, Carlsbad, CA, USA) and submitted for a BLASTn analysis. Sequences were submitted to GenBank under accession number MT790749 (*rrs*), MT855497 (*gyrB*), MT845212 (*flaB*), and MT790750 (IGS).

**Table 1:** Oligonucleotides used for PCR and MLSA of TBRF spirochetes.

Primer	Gene Locus	Sequence (5’-3’)
***rrs***	**16S rRNA**	
UniB†		TACAAGGAGGTGATCCAGC
FD3†		AGAGTTTGATCCTGGCTTAG
16S (+)‡		TACAGGTGCTGCATGGTTGTCG
16S (-)‡		TAGAAGTTCGCCTTCGCCTCTG
Rec4†		ATGCTAGAAACTGCATGA
P10 REV		ACATAAGGGCCATGATGATT
P6 REV		TTTACAGCGTAGACTACCAG
***flaB***	***flagellin***	
flaB-Exp-For		ATGATCATAAATCATAATACGTCAGCTATAAATG
flaB-Exp-Rev		TCTAAGCAATGATAATACATACTGAGGCAC
flaLL§		ACATATTCAGATGCAGACAGAGGT
flaRL§		GCAATCATAGCCATTGCAGATTGT
***gyrB***	***DNA gyrase***	
gyrB 3’§		GGCTCTTGAAACAATAACAGACATCGC
gyrB 5’+3§		GCTGATGCTGATGTTGATGG
***IGS***	***rrs-rrlA IGS***	
IGSF¥		GTATGTTTAGTGAGGGGGGTG
IGSR¥		GGATCATAGCTCAGGTGGTTAG

* For, forward; Rev, reverse.

† Reported by Wang et al [12].

‡ Reported by Porcella et al [13].

§ Reported by Barbour et al [14].

¥ Reported by Bunikis et al [15]

For the phylogenetic analysis three conserved single copy genes were evaluated, *rrs*, glycerophosphodiester phosphodiesterase (*glpQ*), and *flaB*. These genes were used so we could incorporate sequences that were available for the South American species, *Borrelia venezuelensis* [16]. While sequence for *rrs* and *flaB* were produced by PCR and Sanger sequencing (Table 1), we obtained *glpQ* (accession number: MZ229856) after generating the genome of the novel *Borrelia* (manuscript in preparation). GenBank accession numbers were obtained for nine species of relapsing fever spirochete (S1 Table). Nucleotide sequences were first aligned with MAFFT (v7.475) using the auto option and ends were manually trimmed [17–20]. A maximum-likelihood tree was inferred using a concatenation approach in IQ-TREE2 (v2.1.2) with an edge-linked proportional partition model bootstrapped 1,000 times with ultrafast bootstraps [18–20]. Each partition’s substitution model was determined using the MFP option [21]. The MFP option selects the best-fit model from available substitution models that minimizes the Bayesian information criterion score for each partition. Each gene was partitioned individually based on the best-fit model selected by MFP, which were as follows: rrs, HKY+F+I; flaB, HKY+F+G4; glpQ, TIM+F+G4. The resulting tree was visualized using iTOL (v5) and annotated in Inkscape [22]. The tree was rooted on *Borrelia turcica* IST7.

## Results

### *Ornithodoros* collections from field sites in Panama

**Fig 1:**
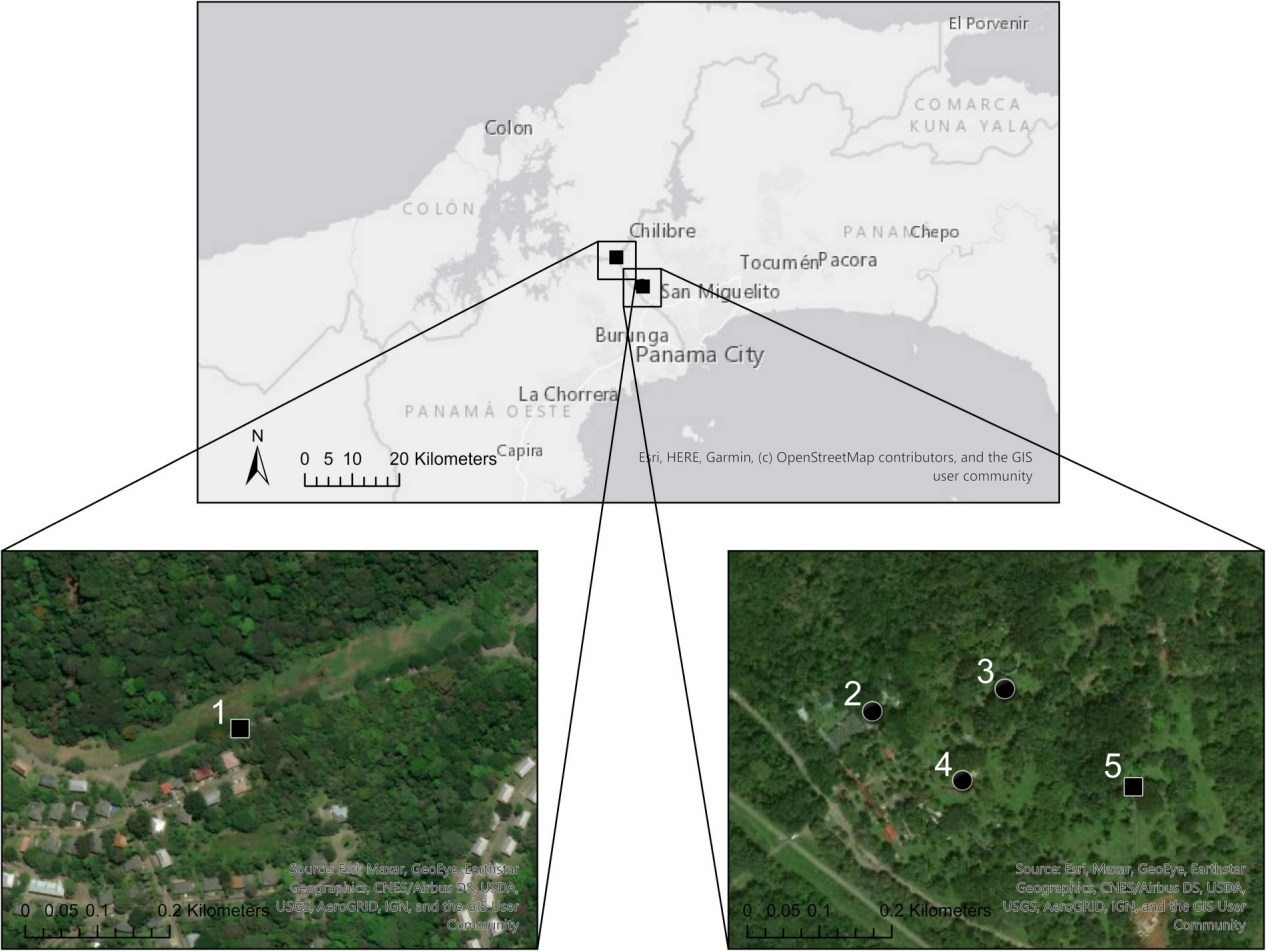
Mapping *Ornithodoros puertoricensis* collection sites. Tick collection efforts were centered in two primary locations (upper insert). The two expanded bottom panels show five sites where collection efforts took place. At sites one and five (squares), either zero ticks were obtained or there were insufficient numbers to test. At sites two through four (circles), ticks were collected. A scale is shown in kilometers in the lower left panels. Information for a given site number is further detailed in Table 2. The greyscale basemap was created using https://services.arcgisonline.com/ArcGIS/rest/services/Canvas/World_Light_Gray_Reference/MapServer, and the imagery basemap was created using https://services.arcgisonline.com/ArcGIS/rest/services/World_Imagery/MapServer.

**Fig 2:**
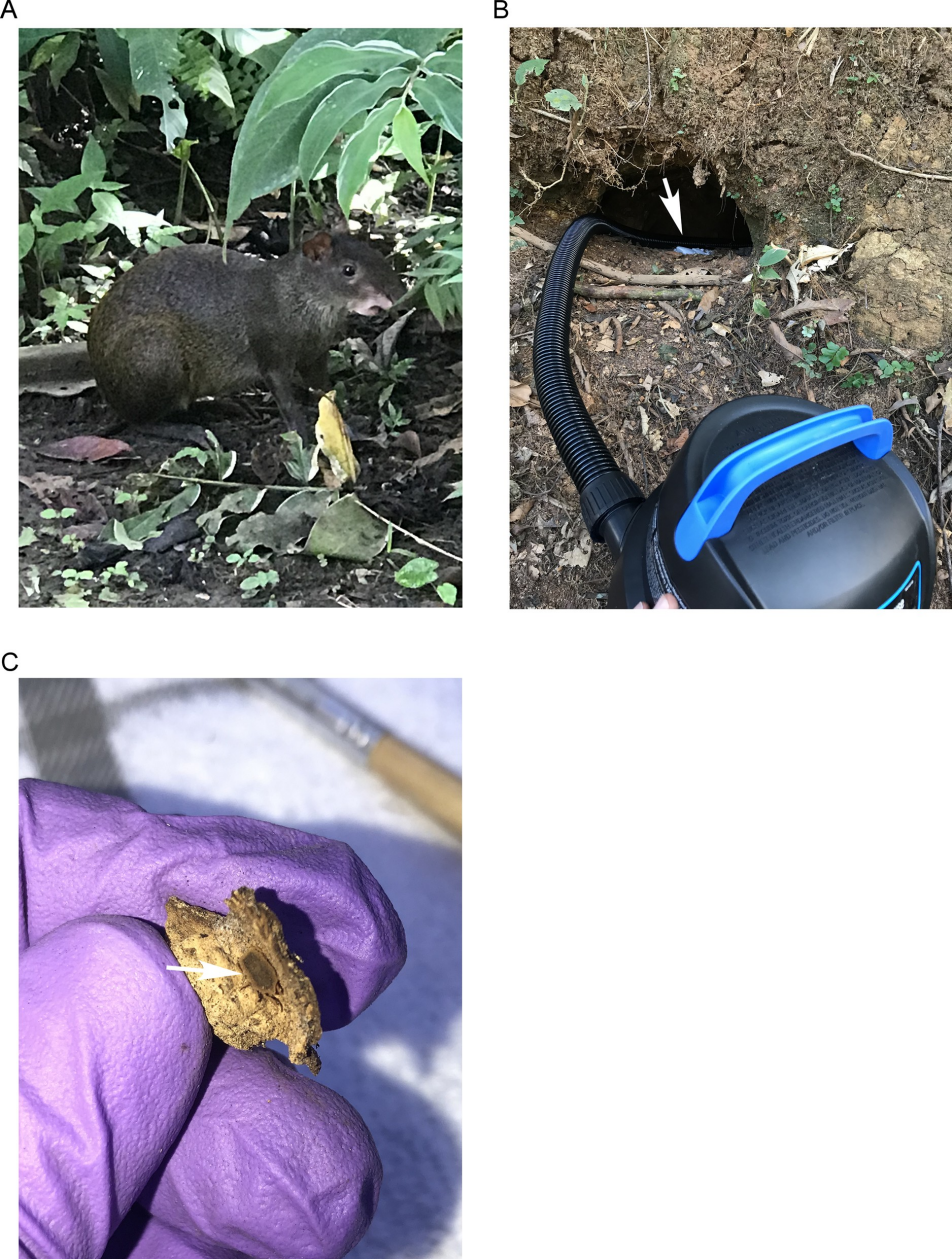
Habitat of *Ornithodoros puertoricensis* collections. At collections sites an abundance of *Dasyprocta punctata* was seen (A) and collection efforts were centered around their dens (B). Two methods were used to collect ticks. Dry ice (B, white arrow) was used as a source of carbon dioxide to lure ticks from within the dens. The dens were also aspirated (B). Debris from dens was evaluated and ticks were observed wedged between pieces of wood (C, white arrow).

Field sites were distributed around Gamboa and in the Ancón community of the City of Panama. Sites included a neighborhood and the botanical garden and zoo of Summit Municipal Park (Fig 1). A primary observation was the abundance and activity of *Dasyprocta punctata*, a large Neotropical rodent (Fig 2A). Given that *D*. *punctata* dig burrows under and around trees, we centered collection efforts around these areas (Fig 2B). Tick collections were also focused in areas where rodent and bird activity (nests and fecal matter) were detected.

Dry ice was used as a source of carbon dioxide to bait ticks and when tick activity was observed within dens an aspirator was used to collect them. As the debris was sifted, argasid ticks were collected from the material. Ticks were frequently visualized wedged within pieces of wood (Fig 2C). Between zero to 32 ticks were collected from a given den (Table 2). In total, ticks were collected from 11 dens. Moreover, while all life stages (larvae, nymphs, and adults) were observed, we only kept nymphs and adult ticks for further analysis.

**Table 2:** Summary of tick collections.

	No. Dens Aspirated	No. Ticks Collected per Den	Tick Life Stage
**Site 1**	3	0	L
**Site 2**	2	5–16	A, N, L
**Site 3**	1	32	A, N, L
**Site 4**	5	0–30	A, N, L
**Site5**	2	0	-

* A: adult, N: nymph, and L: larvae

### Molecular typing of field collected ticks

Since *Ornithodoros* species in Central America are nearly indistinguishable at the nymphal and adult stages, we performed a molecular analysis of the *rrs* locus to speciate collected specimens. Amplification of the *rrs* locus yielded an expected amplicon size of ~470 bp and sequencing and BLASTn analysis identified ~99% identity to *O*. *puertoricensis* previously collected in Panama [6]. These findings supported the identification of *O*. *puertoricensis* in additional regions in the Canal Zone of Panama.

### Isolation and molecular characterization of *Borrelia* colonizing *O*. *puertoricensis*

To determine whether field collected ticks were colonized with TBRF spirochetes, *O*. *puertoricensis* were fed on ICR mice. A drop of blood was evaluated by dark field microscopy for 10 consecutive days, and we failed to visualize spirochetes. However, we detected faint antibody responses in three animals to *B*. *turicatae* protein lysates when using mouse sera that were collected 30 days after feeding ticks on the animals. These antibody responses were stronger than the other two mouse serum samples and we suspected that the animals were infected but the spirochetes failed to reach densities in the blood that would have been detected by microscopy. Consequently, the remaining live ticks from one location were fed again on a MyD88^-/-^ mouse, and within five days spirochetes were visualized in the blood. Spirochetes reached ~1 x 10^8^ spirochetes per ml and were successfully isolated in culture medium.

**Fig 3:**
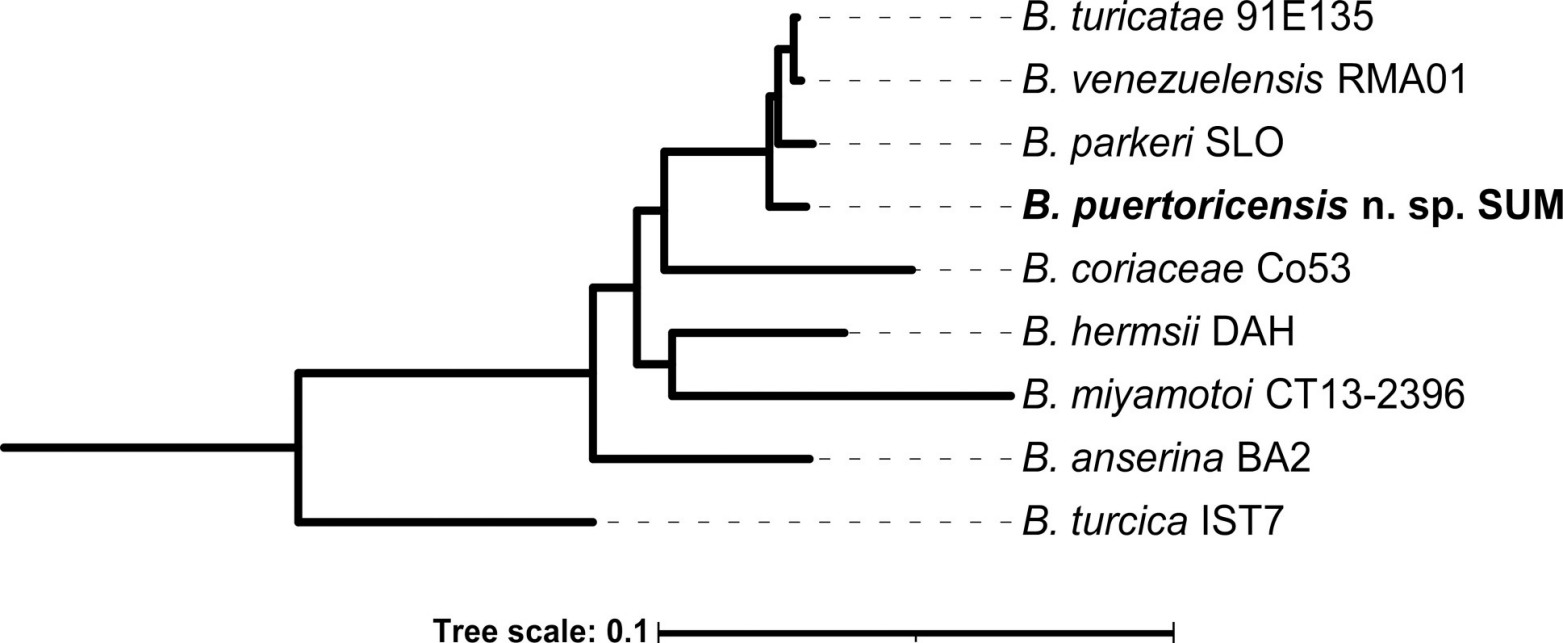
Genetic characterization of *Borrelia puertoricensis* sp. nov. To determine the placement of *B*. *puertoricensis* sp. nov. in relation to other relapsing fever species, a maximum-likelihood phylogenetic tree was inferred by IQ-TREE2 using an edge-linked proportional partition model of alignments of the *rrs*, *glpQ*, and *flaB* sequences. Branch supports are indicated as a percent of 1,000 ultrafast bootstraps. The scale bar shown reflects substitutions per site.

To evaluate the taxonomic position of the *Borrelia* isolated from *O*. *puertoricensis*, four loci were amplified (IGS, *rrs*, *flaB*, and *gyrB*). PCR analysis generated an expected amplicon size for IGS (~635 bp), *rrs* (1,440 bp), *flaB* (~888 bp), and *gyrB* (~520 bp). Sequence and BLASTn analysis of each individual gene indicated that the novel *Borrelia* was most closely related to *B*. *turicatae* and *B*. *parkeri* (89.5–100% nucleotide identity). Furthermore, the phylogenetic analysis using concatenated sequences of *rrs*, *flaB*, and *glpQ* indicated that the novel species of TBRF spirochete was distinct from other species and clustered most closely with *B*. *parkeri* (Fig 3).

**Fig 4:**
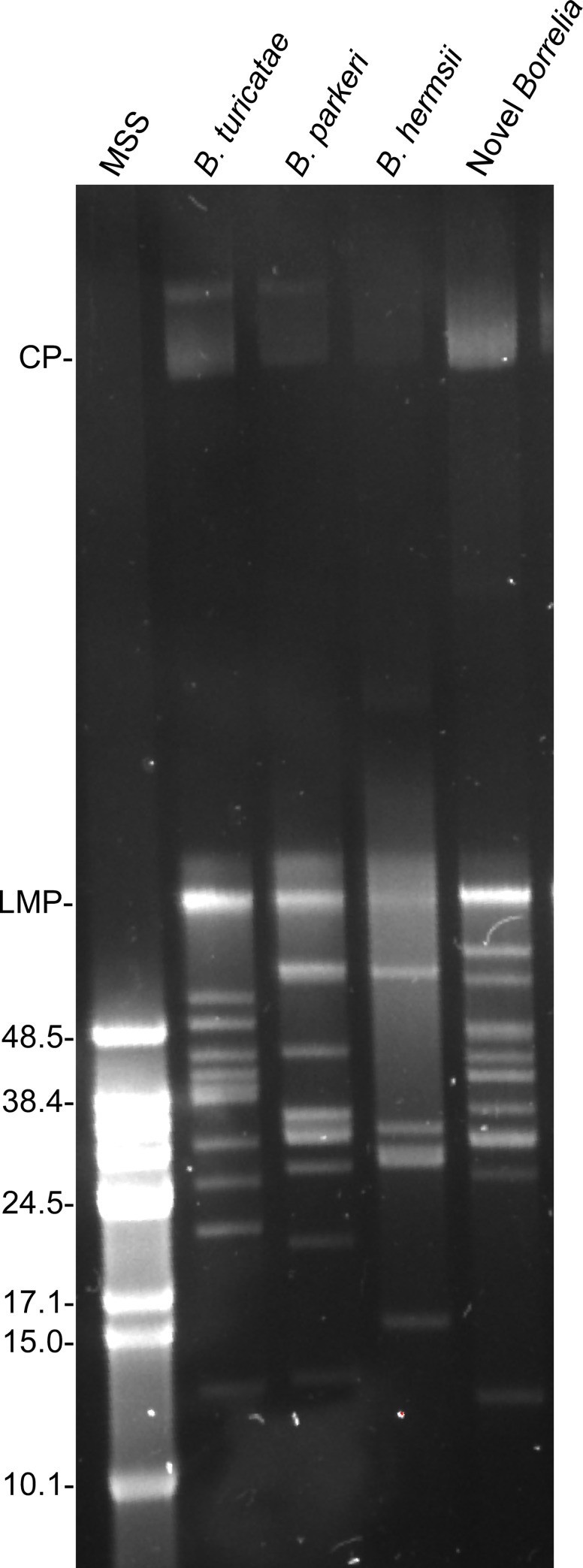
Genomic structure and plasmid analysis of TBRF spirochetes. Pulsed-field electrophoresis was used to assess the genomic structure of the novel *Borrelia* isolated from *O*. *puertoricensis*. Shown at the top of the gel are the species of relapsing fever spirochete used in the analysis. Molecular size standards (MSS) in kilobases are shown on the left. CP: circular plasmid; and LMP: linear megaplasmid.

Performing pulsed-field electrophoresis determined that the *Borrelia* species contained at least 11 linear plasmids (Fig 4). Like the other TBRF spirochete species, we detected a megaplasmid and ~10 linear plasmids ranging in size from ~15–80 kb (Fig 4). Given that this species was isolated from *O*. *puertoricensis* ticks, we propose to use nomenclature consistent with North American *Borrelia* species that were named after their respective tick vector. Therefore, we propose the name *Borrelia puertoricensis* sp. nov. We are in the process of depositing the type strain, *B*. *puertoricensis* SUM, to the American Type Culture Collection and the German Collection of Microorganisms and Cell Cultures.

## Discussion

This study demonstrates the continued circulation of TBRF spirochetes in *Ornithodoros* ticks collected from central Panama, in the former Canal Zone. The collection sites of *O*. *puertoricensis* suggest that vertebrate hosts of these ticks may be small rodents and *Dasyprocta* species, and this was further supported by the observation of *D*. *punctata* in two dens from which ticks were collected. Furthermore, these ticks were infected with spirochetes, and isolation of *B*. *puertoricensis* sp. nov. was achieved in the laboratory. Through genetic characterization, the spirochetes most closely grouped with the North American species *Borrelia parkeri* and contained ~11 linear plasmids.

Previous collections of *O*. *puertoricensis* in regions of Panama spurred our interested to determine whether TBRF spirochetes circulate in the country [6]. Since 2013 more attention has centered on collecting *Ornithodoros* ticks in Panama [6,23,24], and there have been increased reports of *O*. *puertoricensis* colonizing human dwellings from Ancón and Charco La Piedra [25]. Given the longevity of argasids and the ability of TBRF spirochetes to persistently colonize their vector [26], it is not entirely surprising to identify spirochete-infected *O*. *puertoricensis*. While this current study focused collection efforts at a botanical garden and zoo in the Ancón community, middle- and low-income communities surround the field sites and we recommend the evaluation of community exposure to the spirochetes.

This study was centered on obtaining a bacterial isolate from *O*. *puertoricensis* ticks, and we did not determine the prevalence of infected ticks in endemic regions. Our decision to focus on obtaining a spirochete isolate instead of molecular detection of *Borrelia* in ticks was based on inconsistencies of PCR to identify infected ticks. Furthermore, molecular detection of *Borrelia* DNA in field collected ticks does not confirm that the arthropods are competent vectors. However, now that we have isolated a novel *Borrelia* after feeding *O*. *puertoricensis* on mice, molecular prevalence studies on field collected ticks are more valid.

*Ornithodoros puertoricensis* is a well-recognized species in Panama and caution should be taken with the historical records of *Ornithodoros* species in the country because of the challenge associated with speciating these ticks. In the early 1920s the vector of TBRF in Panama was initially described as *Ornithodoros talaje* [2]. This was based on the morphological analysis of nymphs and adult ticks. Concern regarding the speciation of argasid ticks, inducing *O*. *talaje*, began to arise in the early and mid-1900s when new investigations determined several Latin American *Ornithodoros* species were incorrectly designated as *O*. *talaje* [27]. Furthermore, recent work by Venzal and colleagues demonstrated that *O*. *talaje* and *O*. *puertoricensis* are only distinguishable at the larval stage [4]. Clearly, more work is needed to identify *Ornithodoros* species distributed in Panama and capable of transmitting pathogenic TBRF spirochetes to humans.

Our inability to establish an infection in ICR mice after feeding *O*. *puertoricensis* demonstrated the intricacies of host competence for TBRF spirochetes. While most of these species are maintained in rodent-tick life cycles, it is important to identify the particular rodents that serve as competent hosts. This was demonstrated by Burgdorfer and Mavros with the TBRF species, *Borrelia hermsii* [28]. They infected rodents commonly found near locations where human cases of relapsing fever occurred. The animals included *Eutamias amoenus* (chipmunks), *Tamiasciurs hudsoicus richardoni* (pine squirrels), *Glaucomys sabrinus* (flying squirrels), *Spermophilus columbianus columbianus (*Colombian ground squirrels*)*, *Spermophilus lateralis tescorum* (golden-mantled ground squirrels), *Neotoma cinerea cinerea* (wood rats), *Peromyscus maniculatus* (white-footed deer mice), and *Microtus pennsylvanicus* (meadow voles). From these studies, only three species became infected, as determined by the visualization of spirochetes in the blood. In our study, we observed an abundance of *D*. *puncatata* at our field sites and collected *O*. *puertoricensis* from burrows that had been occupied by the animals. However, a limitation in this study was that we did not assess these animals’ in maintaining TBRF spirochetes. Future studies should focus on determining the role of *D*. *puncatata* in the ecology of TBRF.

While the Ancón community of the City of Panama was shown here to be an endemic focus for TBRF spirochetes, additional foci in surrounding Central and South American countries should not be ruled out. Pathogenic spirochetes have been implicated in a case of human disease from a tourist traveling between Guatemala and Belize, while *B*. *venezuelensis* was isolated from argasid ticks recovered in Brazil [16,29]. Given that there are now Central and South American isolates of TBRF spirochetes, comparative genomic studies can be implemented to aid in identifying diagnostic markers for the spirochetes and to clarify the plasmid content of the bacteria. A genomic analysis will also determine whether these species encode the diagnostic antigen, *Borrelia* immunogenic protein A (BipA). We envision that BipA could be used in seroprevalence studies to identify endemic foci and evaluate the exposure of humans and wild and domestic animals to the spirochetes. Moreover, future work should address the geographic range of *B*. *puertoricensis* in Central and South America and focus on the identification of the vertebrate reservoir hosts.

## Supporting information

S1 Table Accession numbers used for the phylogenetic analysis. (DOCX)
