# Supplementary material for: Isolation and genetic characterization of a relapsing fever spirochete isolated from Ornithodoros puertoricensis collected in central Panama
Source: PLoS Negl Trop Dis. 2021 Aug 16;15(8):e0009642. doi: 10.1371/journal.pntd.0009642 (PMC8389842; doi:10.1371/journal.pntd.0009642)
Supplement: S1 Table — (DOCX) [file pntd.0009642.s001.docx]

**S1 Table. Accession numbers used for the phylogenetic analysis.**

|  | Locus | | |
| --- | --- | --- | --- |
| *Borrelia* species | *rrs* | *glpQ* | *flaB* |
|  |  |  |  |
| *B. venezuelensis* RMA01 | MG651649.1 | MG651651.1 | MG651650.1 |
| *B. turicatae* 91E135 | *CP000049.1 | *CP000049.1 | *CP000049.1 |
| *B. puertoricensis* SUM | MT790749 | MZ229856 | MT845212 |
| *B. parkeri* SLO | *NZ_CP005851.2 | *NZ_CP005851.2 | *NZ_CP005851.2 |
| *B. hermsii* DAH | NR_102957.1 | U40762.1 | AY597777.1 |
| *B. coriaceae* Co53 | NR_114544.1 | AF247158.1 | *CP005745.1 |
| *B. anserina* BA2 | *NZ_CP005829.1 | *NZ_CP005829.1 | *NZ_CP005829.1 |
| *B. miyamotoi* CT13-2396 | *NZ_CP017126 | *NZ_CP017126 | *NZ_CP017126 |
| *B. turcica* IST7 | *NZ_CP028884.1 | *NZ_CP028884.1 | *NZ_CP028884.1 |
|  |  |  |  |

* Sequences obtained from whole genome of respective species of *Borrelia* available on GenBank
